# An intramolecular disulphide bond in human 4E-T affects its binding to eIF4E1a protein

**DOI:** 10.1007/s00249-023-01684-7

**Published:** 2023-10-05

**Authors:** Joanna Zuberek, Marek Warzecha, Mateusz Dobrowolski, Anna Modrak-Wojcik

**Affiliations:** Division of Biophysics, Institute of Experimental Physics, Faculty of Physics, University of Warsaw, Warsaw, Poland

**Keywords:** Eukaryotic initiation factor 4E (eIF4E), 4E-transporter (4E-T), Disulphide bond formation, Analytical ultracentrifugation, Thermal shift assay

## Abstract

The cap at the 5’terminus of mRNA is a key determinant of gene expression in eukaryotic cells, which among others is required for cap dependent translation and protects mRNA from degradation. These properties of cap are mediated by several proteins. One of them is 4E-Transporter (4E-T), which plays an important role in translational repression, mRNA decay and P-bodies formation. 4E-T is also one of several proteins that interact with eukaryotic initiation factor 4E (eIF4E), a cap binding protein which is a key component of the translation initiation machinery. The molecular mechanisms underlying the interactions of these two proteins are crucial for mRNA processing. Studying the interactions between human eIF4E1a and the N-terminal fragment of 4E-T that possesses unstructured 4E-binding motifs under non-reducing conditions, we observed that 4E-T preferentially forms an intramolecular disulphide bond. This “disulphide loop” reduces affinity of 4E-T for eIF4E1a by about 300-fold. Considering that only human 4E-T possesses two cysteines located between the 4E binding motifs, we proposed that the disulphide bond may act as a switch to regulate interactions between the two proteins.

**Graphical Abstract:**

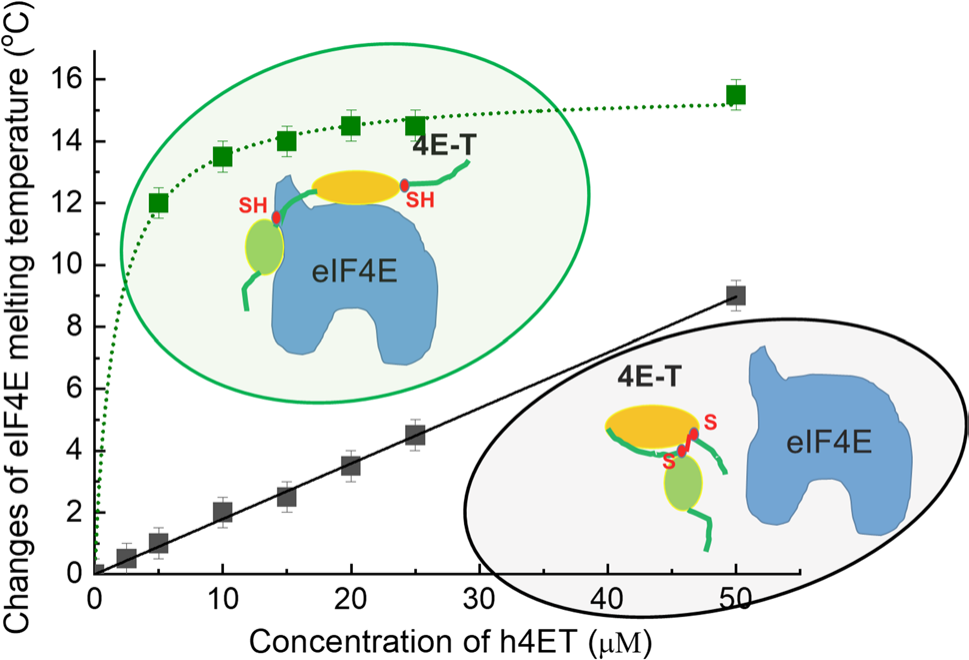

**Supplementary Information:**

The online version contains supplementary material available at 10.1007/s00249-023-01684-7.

## Introduction

The cap structure at the 5′-end of most eukaryotic mRNAs plays a key role in all stages of mRNA metabolism in the cell, beginning from mRNA splicing (Konarska and Padgett 1984; Patzelt et al. 1987), to its nuclear-cytoplasmic export (Culjkovic et al. 2005; Osborne and Borden 2015), to translation initiation and inhibition (Sonenberg and Hinnebusch 2009; Jackson et al. 2010; Ho and Lee 2016), to mRNA degradation in the cell (Li and Kiledjian 2010; Ling et al. 2011). At all these stages, various cap-binding proteins interact with the cap structure and one of these proteins is the eukaryotic translation initiation factor 4E (eIF4E). The cellular functions of eIF4E are connected with the presence of two different binding sites on the protein (Borden 2016): one for the cap at the 5′-end of the mRNA in a narrow hydrophobic slot at the concave part of the protein surface, and a second one for a broad class of 4E interacting proteins (4E-IPs), on the dorsal and lateral surface of eIF4E. The key cellular role of eIF4E is its participation in the formation of the 48S initiation complex during translation initiation (Jackson et al. 2010; Merrick 2015). Binding of eIF4E to the cap at the 5′ end of the mRNA and formation of the trimeric eIF4F complex with the RNA helicase eIF4A and eIF4G proteins, which assembles other complex-forming factors, are the rate limiting step in cap-dependent translation initiation (Sonenberg and Hinnebusch 2009). The interaction of eIF4E with eIF4G is globally negatively regulated by one of the 4E interacting proteins—4E-BP (4E-binding protein) that competes with eIF4G for a common binding site on eIF4E, thereby inhibiting the process of translation initiation (Mader et al. 1995; Richter and Sonenberg 2005). Another 4E-IP protein that acts as a translation repressor is the 4E-Transporter protein (4E-T) (Kamenska et al. 2014a, 2014b, 2016). Moreover, 4E-T was first discovered as a nucleocytoplasmic shuttling 4E-binding protein required for the localisation of eIF4E to the nucleus (Dostie et al. 2000). However, 4E-T is also a component of processing bodies (P-bodies) (Ferraiuolo et al. 2005; Kamenska et al. 2016), cell granules that are formed by liquid–liquid separation of translationally repressed mRNAs and proteins associated with mRNA decay (Luo and Na 2018). In P-bodies, 4E-T, a large disordered protein with multiple regions of low complexity, interacts with several proteins involved in mRNA turnover and acts as a regulator of mRNA storage (Ozgur et al. 2015; Kamenska et al. 2016; Brandmann et al. 2018; Räsch et al. 2020). The protein 4E-T is the factor which brings eIF4E and its isoform 4EHP (eIF4E homologous protein) into P-bodies and promotes repression of translation (Ferraiuolo et al. 2005; Kubacka et al. 2013; Kamenska et al. 2014a, 2016). Moreover, it has been recently shown that the mRNAs bound to 4E-T protein in P-bodies are deadenylated and protected from decapping by interaction of 4E-T with cap-bound eIF4E or 4EHP (Räsch et al. 2020).

4E-T protein, like other non-homologous 4E-IP proteins, contains the canonical eIF4E-binding motif (C-motif) YXXXXLϕ (where X is any amino acid and ϕ is a hydrophobic residue) in its disordered N-terminal region, that interacts with a site on the convex dorsal surface of eIF4E, common to all 4E-IPs (Kamenska et al. 2014b; Hernández 2022). The C-motif of 4E-T, like that of 4E-BP or eIF4G, adopts an α-helical structure upon binding to eIF4E. Downstream of the C-motif, 4E-T possesses the non-canonical eIF4E-binding motif (NC-motif), which has no sequence similarity among 4E-IPs (Igreja et al. 2014). However, it seems that the NC-motif of 4E-IPs binds to the same site on the lateral surface of eIF4E (Peter et al. 2015) (Gruner et al. 2016) forming non-canonical loops*.* The C-motif and the NC-motif are linked by a short region containing the elbow loop which is also important for binding to eIF4E. For *Drosophila melanogaster* (*Dm*) 4E-T, the presence of the NC-motif together with the elbow loop increases its binding to eIF4E about tenfold, compared to the C-motif alone, whereas the NC-motif alone is not sufficient to bind to eIF4E (Igreja et al. 2014). Similar specificity is observed for example in the case of human 4E-BP proteins, but not for Cup protein from *Drosophila melanogaster* (Kinkelin et al. 2012; Igreja et al. 2014).

**Fig. 1:**
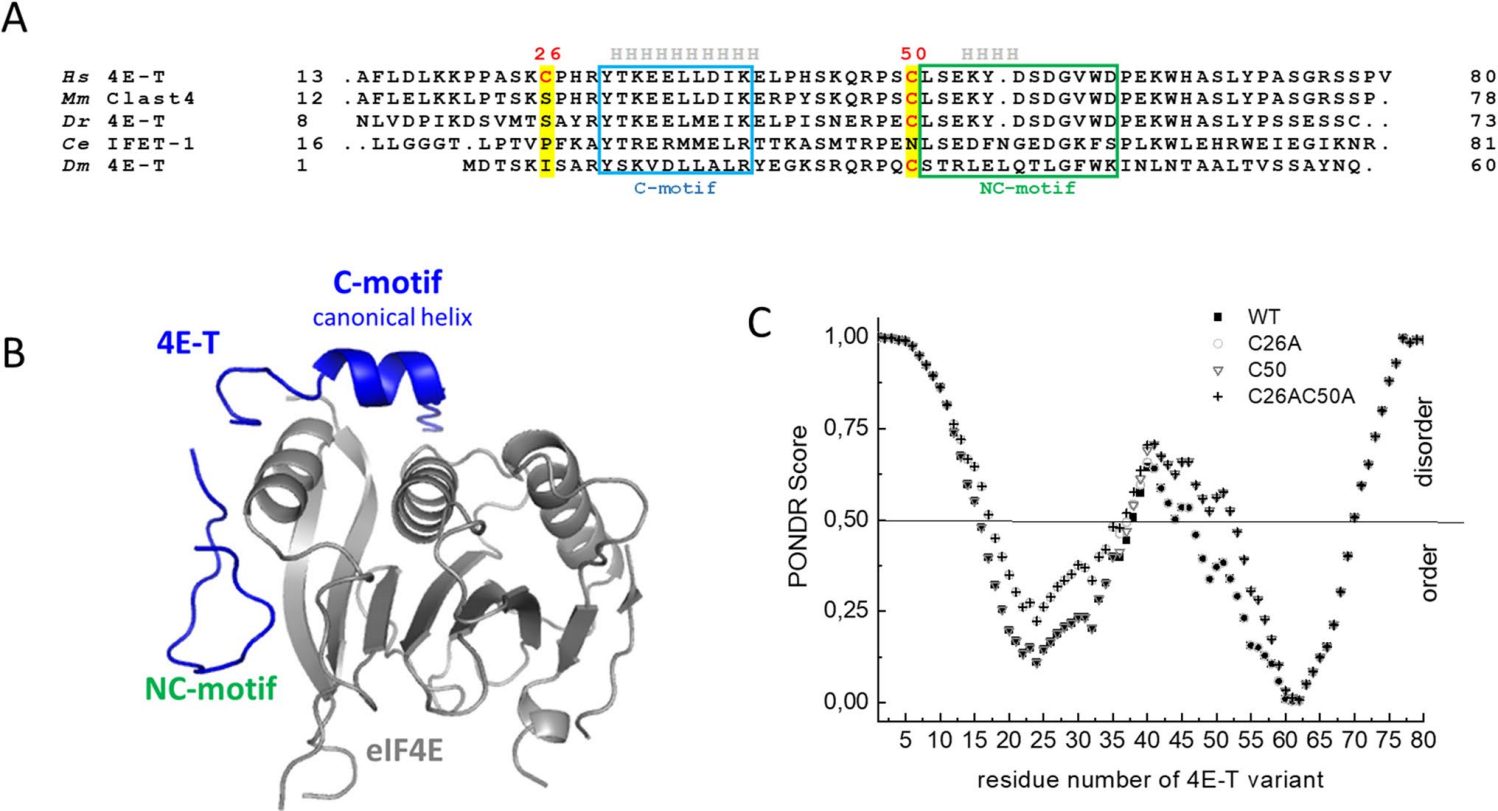
**A** Sequence alignments of eIF4E-binding region in 4E-T proteins from different organism: *Homo sapiens* (*Hs*)*, Mus Musculus* (*Mm*), *Danio rerio* (*Dr*), *Caenorhabditis elegans (Ce*)*, Drosophila melanogaster* (*Dm*). A canonical 4E-binding motif (C-motif) and putative non-canonical motifs (NC-motif) are marked with a box. The position of cysteine residues are marked in red. The regions of 4E-T which form helices upon binding to eIF4E are indicated above the sequences. **B** The crystal structure of complex of *Dm*eIF4E-2 with *Dm*4E-T fragment showing the 4E-binding motifs. PDB code: 4EU9 (Peter et al. 2015). **C** The prediction of disordered regions in N-terminal fragment of human 4E-T protein and its cysteine mutants using PONDR software (Xue et al. 2010)

*Drosophila* 4E-T, like that in other species, possesses a cysteine residue located before the NC-motif, which forms van der Waals contacts with conserved histidine residues of eIF4E as shown in the crystal structure of *Dm* 4E-T with eIF4E (Peter et al. 2015). However, the human 4E-T possesses an additional cysteine residue located before the C-motif (Igreja et al. 2014) (Fig. 1A). To elucidate the role of the additional cysteine in the N-terminal fragment of human 4E-T for the mechanism of 4E-T binding to eIF4E, we prepared single and double cysteine mutants of h4E-T. Furthermore, we examined the ability of h4E-T to form conformations with intra- or intermolecular disulphide bonds and attempted to explain their role in the interaction between human 4E-T and eIF4E.

## Materials and methods

### Cloning and mutagenesis

cDNA of N-fragment human 4E-T(1–68) from pUC57 vector (BIOMATIK) was amplified by PCR and inserted into the pET28a expression vector (Novagen) between NdeI and XhoI sites, to express h4ET(1–68) peptide with the N-terminal His_6_-tag cleavable by thrombin protease.

Site-directed mutagenesis was used to generate cysteine point mutations (C26A, C50A and C26AC50A) of h4E-T(1–68). All constructs were verified by DNA sequencing (Genomed).

### Protein expression and purification

Variants of human 4E-T(1–68) containing the N-terminal His_6_-tag were expressed in *E. coli* Rosseta 2 (DE3)pLysS strain (Novagen). Bacteria were grown in LB medium at 37 °C until OD at 600 nm reached 0.6, and were subsequently induced for 3 h at 37 °C by adding 0.5 mM IPTG. The cells were harvested by centrifugation at 9110×*g* for 15 min, resuspended in lysis buffer (50 mM HEPES–KOH pH 7.2, 300 mM KCl, 20 mM imidazole) containing protease inhibitor mixture without EDTA), and disrupted by sonication. Nucleic acids were removed from the protein sample by incubating the lysate with HS-Nuclease (MoBiTec) for 20 min at 4 °C. The soluble fraction obtained by centrifugation at 43,000×*g* for 30 min was incubated with HIS-Select Nickel Affinity Gel (SIGMA) for 1 h at 4 °C. The resin was then washed with 10 volumes of lysis buffer and protein was eluted with 250 mM imidazole in 50 mM HEPES–KOH (pH 7.2), 300 mM KCl and 2 mM TCEP buffer. Each elution was preceded by a 15-min incubation of the bed in the elution buffer. Imidazole was removed from the protein sample by size-exclusion chromatography on Enrich SEC70 column (Bio-Rad) in 50 mM HEPES–KOH, 100 mM KCl (pH 7.2) or 50 mM HEPES–KOH, 100 mM KCl, 1 mM TCEP (pH 7.2) and fraction corresponding to the monomeric form of the protein was collected. Where required for ITC, DSF and AUC experiments, proteins were concentrated using Amicon Ultra-4 Centrifugal Filters with 3 kDa NMWCO (Merck) and filtrated using Ultrafree MC-HV Centrifugal PVDF filters with 0.45 µm membrane pore (Merck).

Before ITC, DSF and AUC experiments, the buffer in the eIF4E1a samples was exchanged for 50 mM HEPES–KOH, 100 mM KCl (pH 7.2) or 50 mM HEPES–KOH, 100 mM KCl, 1 mM TCEP (pH 7.2) using Amicon Ultra-4 Centrifugal Filters with 10 kDa NMWCO (Merck). Finally, eIF4E1a samples were filtered through using Ultrafree MC-HV Centrifugal PVDF filters with 0.45 µm membrane pore (Merck) to remove protein aggregates.

Protein concentration was determined spectrophotometrically, assuming the theoretical extinction coefficients at 280 nm of 52,940 M^−1^ cm^−1^ for eIF4E1a and 13,980 M^−1^ cm^−1^ for h4E-T(1–68) variants; the extinction coefficients were calculated using ProtParam (Pace et al. 1995).

### Size-exclusion chromatography (SEC)

To determine the oligomeric state of h4E-T variants, size-exclusion chromatography was performed on NGC chromatography system (Bio-Rad) using Enrich SEC70 column (Bio-Rad). Samples of h4E-T variants purified as monomer on SEC70 column, after elution from nickel affinity beads, were incubated in the absence or in the presence of 2 mM TCEP in 50 mM HEPES–KOH (pH 7.2) with different concentrations of KCl for 24 h at 4 °C or 20 °C. After incubation, the samples were loaded onto the column equilibrated with the same buffer without TCEP and eluted with the same buffer at the rate of 0.5 ml/min at 20 °C. The partition coefficient (*K*_AV_) was estimated from the recorded retention volume for the oligomeric state. The calibration curve (*K*_AV_ versus log(MW)) was obtained with proteins from MWG70 KIT (Sigma-Aldrich): bovine serum albumin (66 kDa), carbonic anhydrase from bovine (29 kDa), cytochrome c from horse heart (12.4 kDa), aprotinin from bovine lung (6.5 kDa).

### Analytical ultracentrifugation (AUC)

Analytical ultracentrifugation was run at 20 ºC on Beckman Optima XL-I with 4- or 8-position An-Ti rotor and UV detection at 280 nm in double-sector 1.2 cm cells with charcoal-filled epon centrepieces and sapphire windows. Samples of h4E-T(1–68) variants in 50 mM HEPES–KOH, 100 mM KCl (pH 7.2) with or without 1 mM TCEP were freshly prepared as described above. 390 µl of h4E-T(1–68) solution and 400 µl of the reference buffer were loaded into the right and the left sectors of the cells. Sedimentation velocity experiments were performed at 50,000 rpm. Radial absorption scans of protein concentration profiles were measured at 5 or 8-min intervals. The sedimentation velocity data was analysed using SEDFIT software with a continuous sedimentation coefficient distribution model, c(s), based on the Lamm equation (Schuck 2000). c(s) distributions were integrated to provide the signal weighted average sedimentation coefficients (s). Analysis of the AUC data in SEDFIT also allowed determination of the frictional ratio f/f0 that provides information on the shape of the moving particles. The partial specific volume of h4E-T(1–68) variants (from amino acid composition), as well as the density and viscosity of the buffer, were calculated using the Sednterp software (Laue et al. 1992).

### Differential scanning fluorimetry (DSF)

Differential scanning fluorimetry was performed according to Bio-Rad protocols (Bioradiations 2019) (Niesen et al. 2007) using a CFX96 Real-Time PCR System (Bio-Rad). All reactions were made to a final volume of 25 ul, in DSF buffer (50 mM HEPES–KOH pH 7.2, 100 mM KCl) with or without 1 mM TCEP. The concentration of eIF4E1a protein was 5 µM with increasing concentrations of h4E-T(1–68) variants, from 0 to 50 µM. Prior to measurements, the samples of h4E-T(1–68) variants were incubated for an appropriate time (up to 48 h) in DSF buffer with or without 1 mM TCEP at 4 °C or 20 °C. Control assays were carried out with the buffer alone and with 50 μM solutions of h4E-T(1–68) variants. A temperature gradient from 10 °C to 95 °C was performed at the rate of 1 °C/20 s. SYPRO Orange at a concentration of 5 × was used as a reporter dye to detect protein unfolding. Its fluorescence intensity changes were measured with FRET channel (excitation/emission: 450– 490 nm/560–580 nm).

The resulting data were normalised by converting the raw fluorescence data to a relative percentage of fluorescence according to the formula described previously (Brown et al. 2020). Melting temperature (*T*_m_) was estimated by fitting the Boltzmann equation to the normalised fluorescence data (Niesen et al. 2007). The final *T*_m_ was calculated from a minimum of three independent sets of experiments.

### Isothermal titration calorimetry (ITC)

The ITC experiments were performed at 20 °C using microcalorimeter iTC200 (Microcal, Malvern). Assays were performed under reducing and non-reducing conditions (50 mM HEPES–KOH pH 7.2, 100 mM KCl, with or without 1 mM TCEP). 200 μl of heIF4E1a sample at a concentration of 5 µM or 10 µM placed in the calorimetric cell was titrated with ten-fold concentrated solution of the h4E-T(1–68) variants. The only exception was the titration of eIF4E1a with wild-type human 4E-T(1–68) under non-reducing conditions, where the concentration of eIF4E1a was 20 µM and h4E-T(1–68) WT—200 µM. A single titration experiment consisted of an initial injection of 0.4 µl followed by 28 injections of 1 µl with 180 s intervals, with stirring at 750 rpm. In addition to each ITC experiment, a solution of each h4E-T(1–68) variant was injected into pure buffer to measure the heat of dilution, which was subtracted from the apparent heat of complex formation.

The collected calorimetric data were analyzed using the NITPIC program (Scheuermann and Brautigam 2015) and the thermodynamic parameters were estimated by fitting a one-site model to the obtained isotherms using SEDPHAT software (Zhao et al. 2015).

## Results and discussion

### The presence of Cys26 in human 4E-T has negligible effect on its interaction with eIF4E

In human 4E-T, cysteine 26, which is unique to the human protein, is located before the canonical 4E-binding motif (C-motif) and a conserved cysteine 50 is located close to the non-canonical motif (NC-motif) (Fig. 1A). The N-terminus of 4E-T is intrinsically disordered and within the C-motif an α-helix forms as a result of interaction with eIF4E protein (Fig. 1A, B). First, the potential influence of cysteine residues on the formation of structured regions at the N-terminus fragment of 4E-T was analysed using a meta-predictor of natural disordered regions –PONDR software (Xue et al. 2010). The substitution of both cysteine residues by alanine slightly disrupts the ordered regions corresponding to the binding motifs, the C-motif in the case of C26 and the NC-motif in the case of C50 (Fig. 1C).

**Fig. 2:**
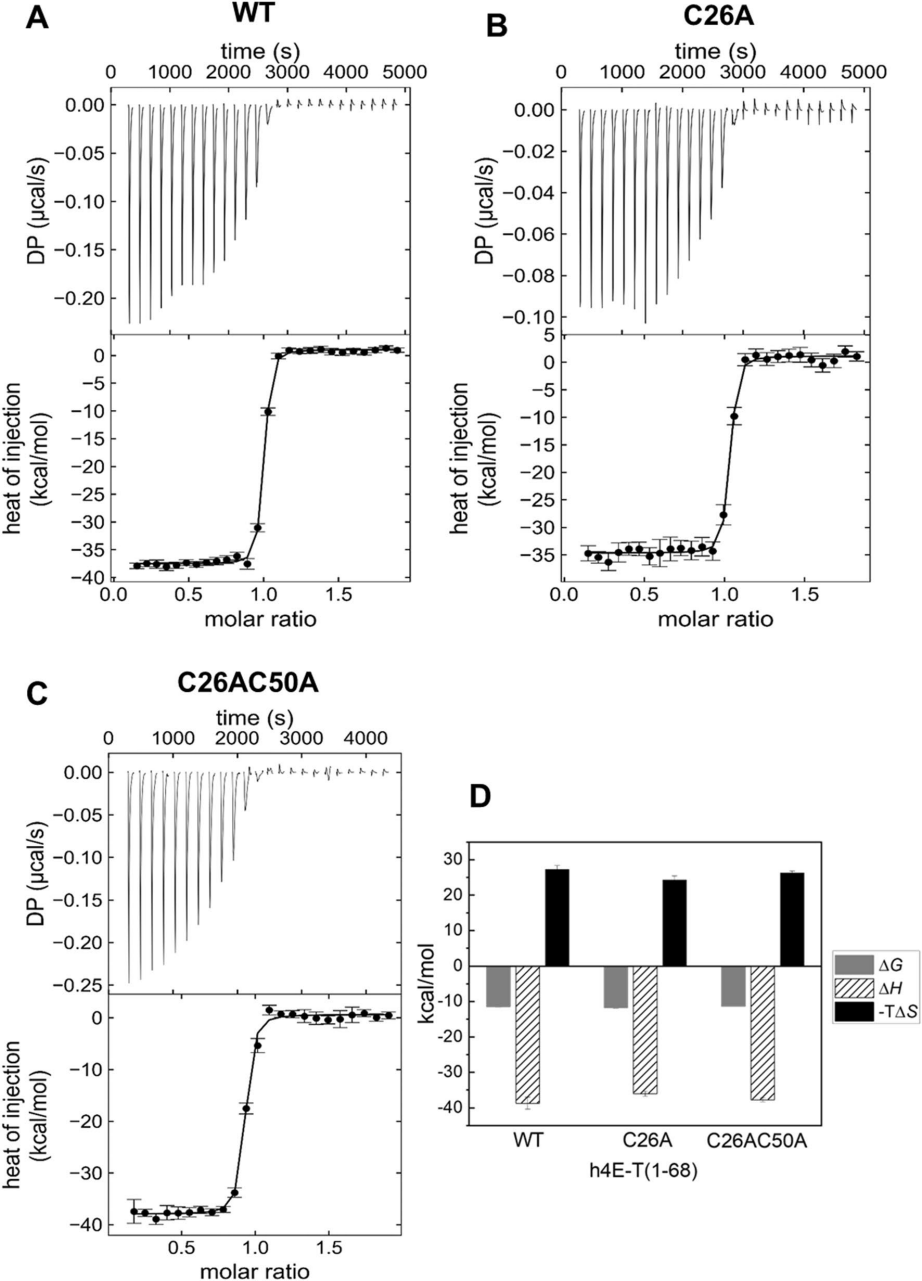
Thermodynamic profile of binding of human eIF4E1a to h4E-T variants: (**A**) WT, (**B**) C26A mutant, (**C**) C26AC50A mutant based on isothermal titration calorimetry (ITC) in the presence of 1 mM TCEP. The measured parameters include the Gibbs free energy of binding (Δ*G*), the enthalpy change (Δ*H*) and temperature dependent entropy change (*− T*ΔS) (**D**). The titrations were performed when aliquotes 50 µM of the h4E-T variant were injected to the calorimetric cell containing 200 µl of eIF4E1a at a concentration of 5 µM

Next, binding affinities and thermodynamic parameters of complex formation between eIF4E and 4E-T variants were measured by isothermal calorimetric titration experiments (Fig. 2). The ITC assays were performed in a buffer containing1 mM TCEP to prevent the formation of disulphide bonds between cysteine residues. The dissociation constants obtained for cysteine mutants are similar to each other and to those for wild type protein (Table 1). The N-terminal fragment of 4E-T binds to eIF4E with very high affinity, with *K*_D_ of 2.7 ± 0.5 nM for the wild-type protein, which is comparable to the binding constant previously reported for the *Drosophila* proteins (Igreja et al. 2014). The double cysteine mutant C26AC50A binds to eIF4E with *K*_D_ of 3.1 ± 0.2 nM, whereas mutant C26A has a slightly higher binding affinity for eIF4E with *K*_D_ of 1.5 ± 0.5 nM. This may indicate that cysteine 26 has a slightly negative effect on 4E-T binding to eIF4E or that the substituted alanine has a positive effect. In the case of the double mutant, the positive effect of the absence of cysteine 26 is compensated by the negative effect of the absence of cysteine 50, since the latter stabilizes the binding of the non-canonical motif to the lateral surface of eIF4E, as shown by structural studies (Peter et al. 2015). Furthermore, the interaction of all 4E-T variants with eIF4E is enthalpically-favourable and entropy-opposed at 20 °C (Fig. 2D; Supplementary Table 2). The negative enthalpy contribution means that binding is driven by formation of a significant number of hydrogen bonds and non-covalent interactions. On the other hand, the unfavourable entropy change points to a loss of conformational freedom most likely due to the formation of α-helices within the 4E-binding motif of 4E-T. However, the unfavourable entropy contribution for binding of the C26 mutant to eIF4E is slightly smaller than for other 4E-T variants. These results may be related to the positive effect of cysteine 26 on the ordering of the canonical binding motif, as demonstrated by an analysis predicting the disordered regions at the N-terminus of 4E-T (Fig. 1C).

**Table 1:** Parameters for the interaction of human eIF4E1a protein with 4E-T(1–68) variants in the absence and presence of TCEP

Variant of h4E-T(1–68)	TCEP	Data from ITC *K*_D_ (nM)	Data from DSF Δ*T*_m_ (^o^C)	
			Ratio eIF4E:h4E-T(1–68)	
			1:1	1:10
WT	−	800 ± 96	1.0 ± 0.5	9.5 ± 0.5
	+	2.7 ± 0.5	12.0 ± 0.5	15.5 ± 0.5
C26A	−	nd^a^	9.0 ± 0.5	12.5 ± 0.5
	+	1.5 ± 0.5	12.0 ± 0.5	15.5 ± 0.5
C50A	−	nd	10.5 ± 0.5	14.5 ± 0.5
	+	nd	11.5 ± 0.5	15.5 ± 0.5
C26AC50A	−	3.1 ± 0.2	10.5 ± 0.5	14.5 ± 0.5

^a^The sample contains mixture of monomeric and dimeric forms

### Oligomeric forms of cysteine variants of the N-terminal h4E-T fragment

The ability of cysteine residues to form disulphide bonds is significantly influenced by their spatial accessibility and proximity, the differences between the p*K*_a_ of the involved SH groups, the pH of the environment and the redox conditions. The stability of an intramolecular S–S bond in peptides or proteins is proportional to the number of residues forming the “disulphide loop”, located between the linked cysteines (Rajpal and Arvan 2013). In contrast to proteins from other organisms, the N-terminus of human 4E-T possesses two cysteine residues at a distance of 23 amino acids in the sequence within the eIF4E binding motifs (Fig. 1A). In addition, this 4E-T fragment is disordered until eIF4E binds. These factors may favour the formation of inter- or intramolecular disulphide bonds in the case of human 4E-T(1–68) peptide.

**Fig. 3:**
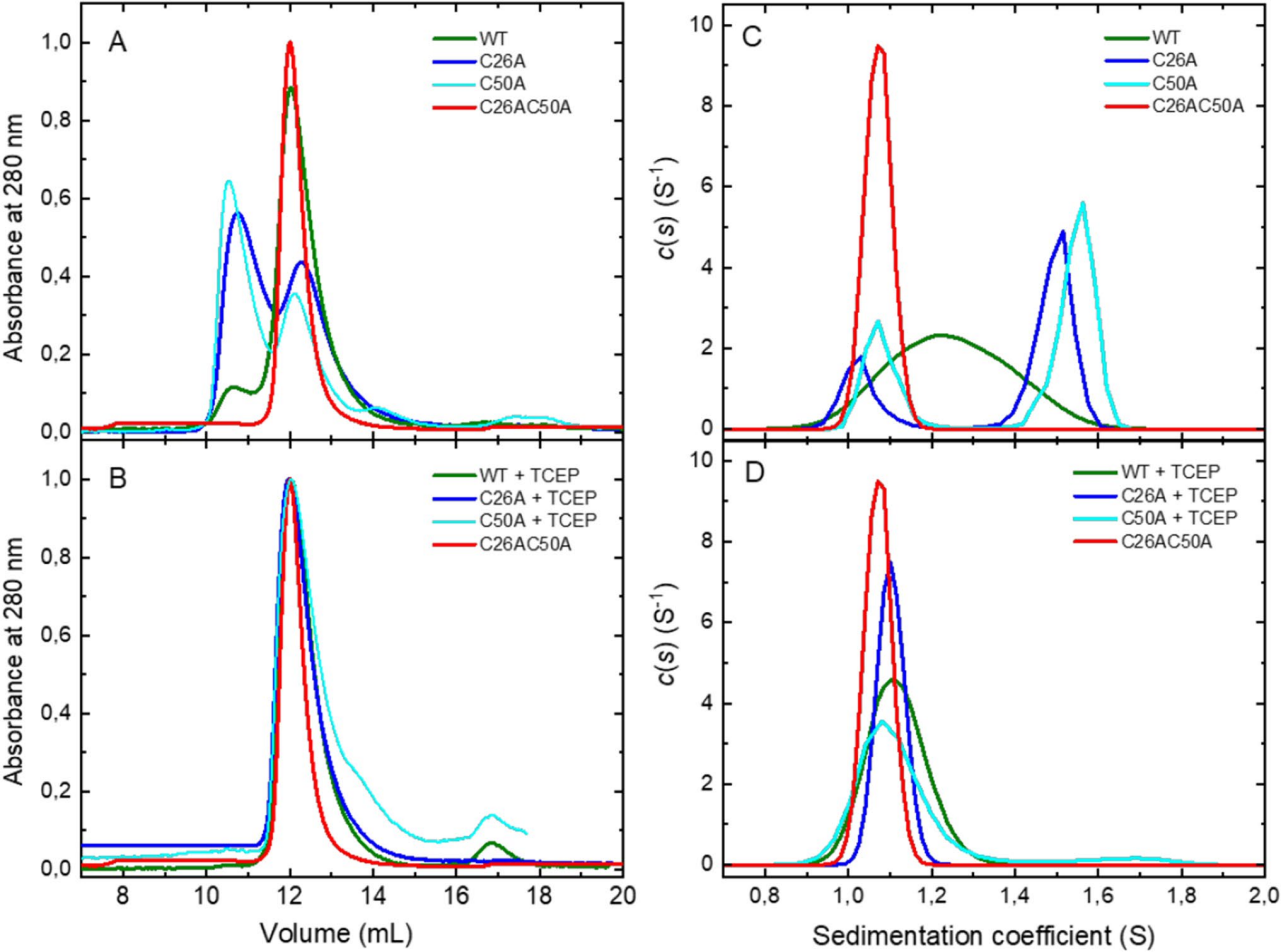
Oligomeric profile analysis. Size exclusion chromatograms (**A, B**) and (**C**, **D**) sedimentation coefficient distribution *c*(*s*) of the h4E-T(1–68) variants in the absence and presence of TCEP showing the oligomeric state. The samples of h4E-T(1–68) variants, after elution from Ni–NTA gel, were purified on SEC column as the monomer fraction and next analysed again by SEC or AUC experiments. During the AUC experiments the samples concentration was 50 µM for WT, C50A and C26AC50A variants and 38 µM for C26A variant

To investigate what type of disulphide bonds (inter- or intramolecular) and thus what oligomeric forms are taken by the human 4E-T(1–68) protein, size exclusion chromatography was performed under non-reducing conditions. For comparison, the single cysteine mutants (C26A, C50A) able to form only the dimer and the double cysteine mutant (C26AC50A) unable to form any disulphide bond were analysed. The samples of h4E-T variants eluted from NTA column under reducing conditions (2 mM TCEP) were purified by size exclusion chromatography under non-reducing conditions. Next, the collected monomeric fractions were incubated in TCEP-free buffer at 20 °C for 24 h and separated by size-exclusion chromatography (Fig. 3A). Size-exclusion chromatography of the double cysteine mutant (C26AC50A) showed the expected monomeric state. For the single cysteine mutants, two peaks were observed on the chromatogram with retention volumes corresponding to the masses of the dimeric and monomeric forms (Table 2; Fig. 3A). For both cysteine mutants, the dimer is the dominant form, with the dimer to monomer ratio of about 60% to 40%. In contrast, for the wild-type protein, a small fraction corresponding to the dimeric state was observed (about 10%), while the monomeric form was the predominant one (90%). The SDS-PAGE analysis in the absence of reducing agent confirmed the observed distribution of oligomeric forms (Supplementary Fig. 1S). Taking into account the above-mentioned factors promoting the formation of the “disulphide loop” in the wild-type 4E-T, it is possible that the monomeric fraction observed by SEC corresponds to a monomer with intramolecular S–S bond (monomer SS) or to a mixture of two different monomeric forms—monomer SS and monomer SH (with both cysteines in reduced state). The control SEC analysis of all 4E-T variants incubated under reducing conditions (2 mM TCEP) showed that all proteins were in the monomeric SH form (Fig. 3B), with retention volumes corresponding to their masses (Table 2).

**Table 2:** Summary of size exclusion chromatography and sedimentation velocity experiments for human 4E-T(1–68) variants in the absence and presence of TCEP

Variants of h4E-T (1–68)	Molecular mass (kDa)	TCEP	Data from SEC		*f/f*_*o*_	Data from AUC			
			Molecular mass (kDa)			*s*(S) (content %)		Molecular mass (kDa)	
			Peak 1	Peak 2		Peak 1	Peak 2	Peak 1	Peak 2
WT	10.065	–	24.2 ± 1.8	9.6 ± 0.7	1.447	1.25 ± 0.14	–	11.3	–
		+	–	9.7 ± 0.7	1.573	1.12 ± 0.07	–	10.5	–
C26A	10.033	–	23.4 ± 1.8	8.1 ± 0.6	1.536	1.03 ± 0.05	1.49 ± 0.05	9.1	15.8
		+	–	10.0 ± 0.7	1.578	1.10 ± 0.03	–	10.5	–
C50A	10.033	–	26.9 ± 2.0	9.0 ± 0.7	1.382	1.08 ± 0.05	1.55 ± 0.05	8.9	15.3
		+	–	9.6 ± 0.7	1.449	1.11 ± 0.09	1.65 ± 0.10	9.9	18.1
C26AC50A	10.001	–	–	10.0 ± 0.7	1.574	1.07 ± 0.03	–	10.5	–

To clarify whether h4E-T forms the monomer SS, AUC experiments under reducing and non-reducing conditions were performed. Sedimentation velocity experiments under reducing conditions (1 mM TCEP) showed that the wild-type h4ET(1–68) and single cysteine mutant C26A sediment as a single species with sedimentation coefficients of ~ 1.1 S, which correspond to monomeric forms (Fig. 3D; Table 2). In the case of the C50A mutant, a small amount of dimeric form with *s* = 1.7 S (5%) is detected. The high values of frictional ratio *f*/*f*_0_ (~ 1.5–1.6) for all the above variants confirm that the proteins are partially unfolded in solution. Single peaks are also present in the *c*(*s*) distributions of WT and C26AC50A variants under non-reducing conditions (Fig. 3C). The *c*(s) distribution of C26AC50A mutant is similar to the distributions obtained with 1 mM TCEP for wild-type protein and single cysteine mutants (Fig. 3C, D). For wild-type h4ET(1–68) without TCEP, however, the peak is very broad indicating that at least two types of structures are in rapid equilibrium (Schuck 2003). Moreover, the *c*(*s*) maximum occurs at higher value than under reducing condition (1.25 S vs 1.12 S) and the value of *f*/*f*_0_ is lower (1.48 vs 1.57) (Table 2). The results suggest that WT h4ET(1–68) creates an intramolecular disulphide bond, which makes the protein molecule more globular (lower *f*/*f*_0_), and leads to faster sedimentation. Under the same condition the C26A and C50A mutants, which can only form intermolecular S–S bonds, sediment as a mixture of monomers (~ 30%) and dimers (~ 70%).

In conclusion, both SEC and AUC experiments showed that under non-reducing conditions wild-type h4E-T preferentially creates intramolecular S–S bonds and that the formation of intramolecular S–S bond prevents the formation of dimers.

### Non-reducing conditions significantly weaken the ability of wild-type h4E-T to bind eIF4E

Disulphide bonds are important structural elements that stabilise protein structures. However, unlike peptide bonds, the disulphide bonds are reversible and as recently found they can be regulatory elements influencing protein activity. They can act like switches to induce changes of protein function, its affinity for a ligand or a protein partner (Passam and Chiu 2019).

To elucidate the potential regulatory function of the “disulphide loop” formation in human 4E-T, binding studies with eIF4E under non-reducing conditions were performed. Considering that samples of 4E-T variants in the absence of a reducing agent are a mixture of appropriate oligomeric forms (see above), the fluorescence-base thermal shift method was used to study the interaction between proteins, instead of ITC.

This technique, called also differential scanning fluorimetry (DSF) is a method that allows the study of changes in protein thermostability as a result of ligand binding. The DSF method enables monitoring of thermal protein unfolding by recording changes in the fluorescence of a dye that binds to the hydrophobic regions of a protein exposed during denaturation (Niesen et al. 2007; Senisterra et al. 2012; Gao et al. 2020). The midpoint of the thermal melting curve recorded using a real-time PCR instrument corresponds to the temperature at which half of the protein is in the unfolded state. This temperature is designated as the melting temperature (*T*_m_) and determines the thermal stability of the protein. Binding of a ligand or an unstructured peptide to a protein can increase its thermal stability and shift its melting temperature. Differences between melting temperatures of a protein in the absence and presence of increasing concentrations of a ligand (Δ*T*_m_) correlate to the binding affinity, as shown previously (Niesen et al. 2007; Senisterra et al. 2012; Krishna et al. 2013; Brown et al. 2020).

**Fig. 4:**
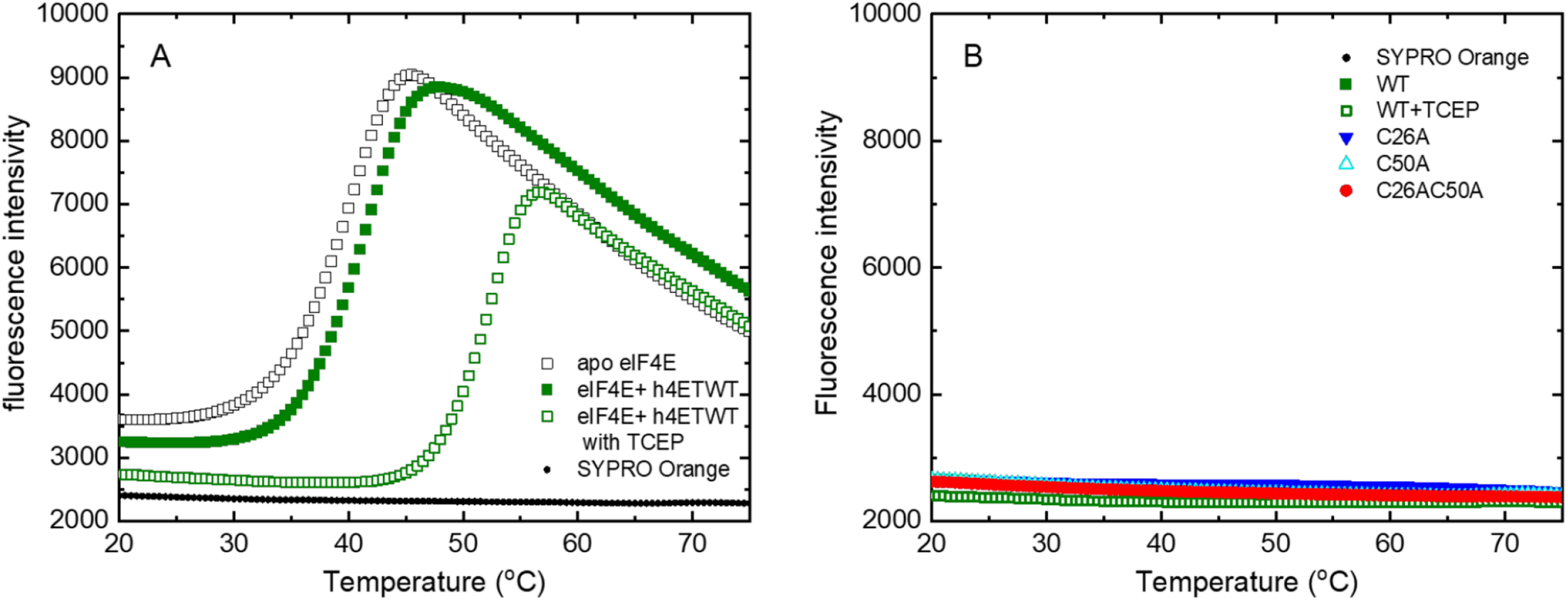
Thermal denaturation assays using DSF method. **A** The representative melting curves of eIF4E (5 µM) in the absence or presence of wild-type human 4E-T(1–68) (5 µM) in 50 mM HEPES–KOH, 100 mM KCl, with or without 1 mM TCEP. With protein unfolding, the SYPRO Orange dye binds to exposed hydrophobic region of protein increasing its fluorescence intensity. The minimum fluorescence is observed for the folded state of protein and maximum fluorescence is observed for the unfolded state. **B** The fluorescence of SYPRO Orange dye alone and in the presence of h4E-T(1–68) variants upon increasing temperature

eIF4E is a globular protein which exhibits a two-phase denaturation transition with *T*_m_ of 40.5 ± 0.5 °C and its interaction with h4E-T causes thermal shifts to higher values (Fig. 4A). The performed analysis also showed that the N-terminus of human 4E-T itself, regardless of the oligomeric form, does not give a signal above the base fluorescence of the free dye. (Fig. 4B) Therefore, in DSF experiments, 4E-T peptide can be treated as a low molecular weight ligand.

**Fig. 5:**
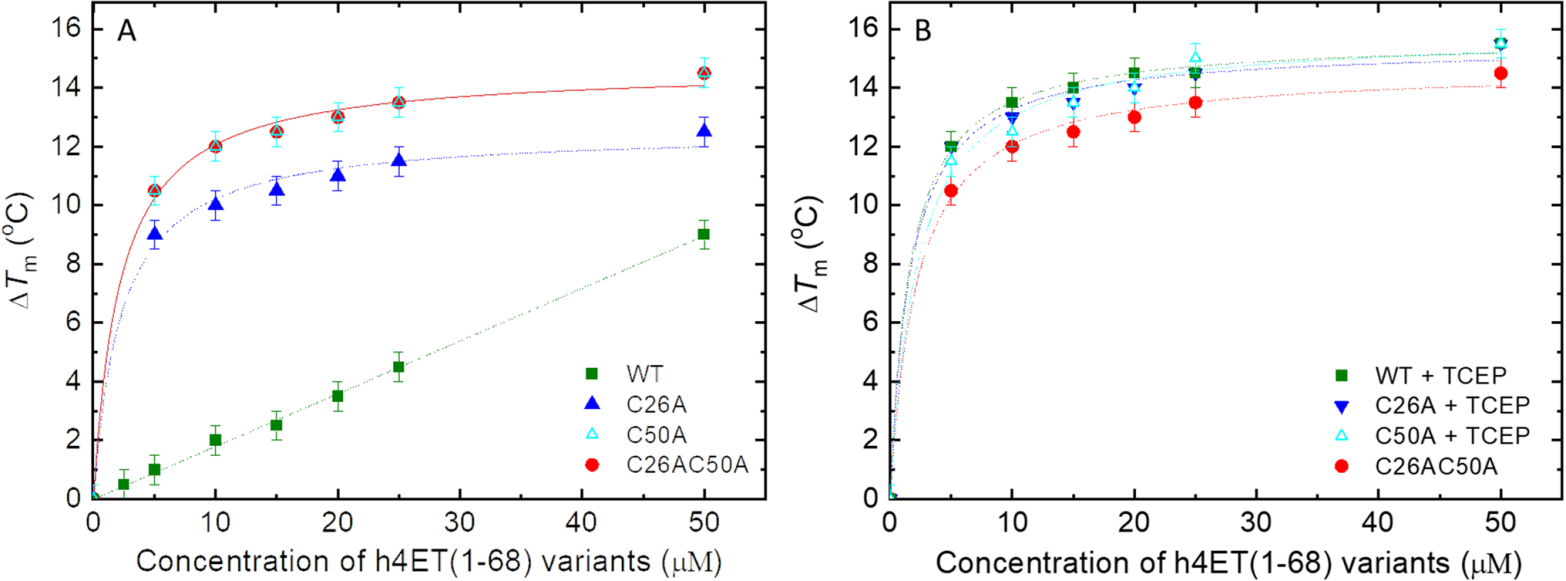
Concertation-dependent thermostability of human eIF4E1a protein by variants of human 4E-T(1–68) fragment in the absence (**A**) or presence (**B**) of TCEP. The melting temperature (*T*_m_) for 5 µM of heIF4E1a was measured by DSF for the apo form and at the increased concentration of h4E-T variants from 1 µM to 50 µM in 50 mM HEPES–KOH pH 7.2, 100 mM KCl with 1 mM TCEP or without. The samples of h4E-T(1–68) variants were incubated for 48 h in 50 mM HEPES–KOH pH 7.2, 100 mM KCl at 4 °C (**A**) and for 24 h in 50 mM HEPES–KOH pH 7.2, 100 mM KCl with 1 mM TCEP at 4 °C (**B**). The curves represent a Hill model fitted to the changes of eIF4E1a’s *T*_m_ values(Δ*T*_m_) against h4E-T(1–68) concentration

Analysis of thermal stability of eIF4E in the presence of increasing concentrations of human 4E-T variants showed that for all 4E-T variants except the double cysteine mutant stabilisation of eIF4E is weaker under non-reducing conditions as compared to reducing conditions. (Fig. 5). The wild-type h4E-T, which is able to form an intramolecular disulphide bond, stabilises eIF4E most weakly. The change of eIF4E melting temperature (Δ*T*_m_) in the 1:1 mixture is only 1.0 ± 0.5 °C, whereas in the presence of TCEP it is 12.0 ± 0.5 °C (Table 1). The single cysteine mutants exhibit high differences in Δ*T*_m_. In the absence of TCEP, Δ*T*_m_ is 9.0 ± 0.5 °C for the C26A mutant and 10.5 ± 0.5 °C for the C50A mutant, as compared to Δ*T*_m_ of 12.0 ± 0.5 °C in the presence of TCEP for both proteins. These differences in the potential for thermal stabilisation by the single cysteine mutants may result from different conformations of dimers and their different affinity for eIF4E, as well as differences in the monomer to dimer ratio in the samples. However, these results clearly demonstrated that the dimeric forms of 4E-T may bind to eIF4E efficiently.

Summing up, the DSF experiments showed that the intramolecular disulphide bond, and not the intermolecular bond, significantly weakens the ability of 4E-T to bind to eIF4E. Moreover, the very poor thermal stabilization of eIF4E by wild-type h4E-T under non-reducing conditions indicates that most of the protein is probably in the monomeric SS form. To confirm this observation, the dissociation constant (*K*_D_) for the complex of eIF4E with wild-type h4E-T(1–68) in the absence of TCEP was determined by ITC (Table 1). The obtained *K*_D_ = 800 ± 96 nM is about 300-fold higher than the value observed under reducing conditions (*K*_D_ = 2.7 ± 0.5 nM). Furthermore, in this case both enthalpy and entropy negative contributions are not very high (Supplementary Table S2). This may indicate that the “disulphide loop” probably closes accessibility to 4E-binding sites and/or prevents the formation of α-helices, which are important for the interaction with eIF4E.

### Dynamics of intramolecular S–S bond formation in h4E-T

Spontaneous disulphide bond formation in vitro is a time-dependent process that is strictly reliant on the environmental conditions such as pH, ionic strength or temperature and on relative position of cysteines. Sedimentation velocity experiments under non-reducing conditions showed that the *c*(*s*) distribution of the freshly purified wild-type h4ET(1–68) sample is characterised by a very broad peak (Fig. 3C). This indicates that two forms of h4E-T monomer SH and monomer SS are in rapid equilibrium on the timescale of the ultracentrifugation process (several hours). Therefore, considering the much higher affinity of h4ET(1–68) for eIF4E (Table 1) and the significantly stronger thermal stabilisation of eIF4E by 4E-T in the presence of TCEP (ΔΔ*T*_m_ = 11.0 ± 0.7 °C, Table1), the DSF method was chosen as a rapid measurement technique to investigate the time scale of the intramolecular disulphide bond formation.

The sample of h4E-T(1–68) WT eluted from the NTA column under reducing conditions (2 mM TCEP) was purified by size exclusion chromatography under non-reducing conditions. The collected monomeric fraction was then incubated at 4 °C or 20 °C for a specified period of time. At defined time intervals, the ability of h4E-T to thermostabilise eIF4E in a 1:1 mixture was investigated and the *T*_m_ of eIF4E in complex with h4E-T was determined. Analogous assays were performed for the monomeric form of h4ET incubated in a buffer with TCEP.

**Fig. 6:**
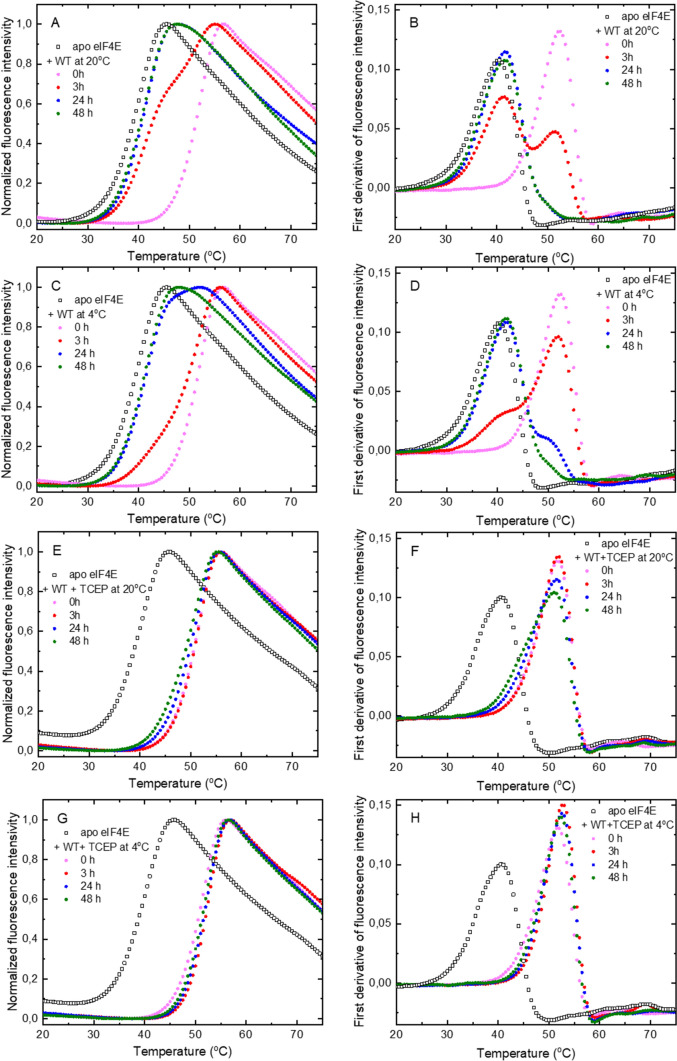
Time-scale analysis of the formation of the intramolecular disulphide bond in h4ET(1–68). The thermal denaturation curves of eIF4E without or with h4ET(1–68) samples incubated in buffer: without TCEP at 20 °C (**A**, **B**) and 4 °C (**C**, **D**) with 1 mM TCEP at 20 °C (**E**, **F**) and 4 °C (**G, H**) measured by DSF. First derivatives of the thermal denaturation curves show clearly the *T*_m_ shift upon formation of the intramolecular disulphide bond in h4ET(1–68) for the samples incubated without TCEP (**B**, **D**). The assays were performed for 5 µM eIF4E in the absence or presence 5 µM wild type h4E-T(1–68)

The obtained thermal denaturation profiles of eIF4E samples in the apo form and in the presence of the appropriate sample of h4E-T(1–68) are shown in Fig. 6. The melting curves are presented as relative fluorescence intensity of SYPRO Orange versus temperature and as an alternative representation using the first derivative showing the melting peaks, where *T*_m_ corresponds to the apex. At “zero” time, all the denaturation profiles for the heIF4E1a:h4E-T(1–68) mixture are similar for all samples. All mixtures exhibit the same *T*_m_ value of 52.5 ± 0.5 °C (for comparison *T*_m_ of the eIF4E apo form is 40.5 ± 0.5 °C). These results clearly show that at “zero” time all samples of 4E-T(1–68) with or without TCEP contain the same oligomeric form i.e. monomer SH. Moreover, under experimental conditions, most eIF4E molecules are bound with 4E-T(1–68), since the estimated dissociation constant (*K*_D_) for the monomer SH is 2.7 ± 0.5 nM (Table 1). For 4E-T samples in the TCEP buffer, further incubation does not alter the thermal denaturation profiles, whereas for samples without TCEP, a gradual thermal shift to a lower *T*_m_ is observed. After 3 h of incubation at 20 °C, two melting peaks are observed: the first, with a *T*_m_ of of 51.5 ± 0.5 °C, corresponding to the monomer SH and the second, a new one, with a *T*_m_ of of 41.5 ± 0.5 °C associated with the monomer SS. The ratio of melting peak heights indicates the amount of each form in the sample. After 24 h of incubation at 20 °C, only one melting peak corresponding to the monomer SS is observed. At 4 °C the formation of the intramolecular disulphide bond is much slower. After 3 h, only a small amount of protein is in the monomeric SS form. After 24 h of incubation, the major form of 4E-T(1–68) is the monomer SS, but a small amount of the monomer SH is still detected by the DSF as evidenced by a small right arm of the melting peak near 50 °C.

Generally, the obtained data showed that under non-reducing conditions, the amount of the monomer SS in 4E-T(1–68) samples gradually increases up to the level where it is the dominant fraction. A different effect, as demonstrated by SEC and AUC studies (Fig. 3A, C), is observed for the single cysteine mutants. Even after 48 h of incubation, the dimer/ monomer ratio is approximately 60% to 40%.

### Monomeric SS form, not dimeric form, of 4E-T blocks interaction with eIF4E

The analysis of the thermal stabilisation of eIF4E by h4E-T(1–68) cysteine mutants under non-reducing conditions presented above was performed on h4E-T cysteine mutant fractions containing a mixture of monomeric and dimeric forms. (Fig. 5A). It is important to know the actual impact of h4E-T dimerization on complex formation with eIF4E. After 24 h incubation of the cysteine mutants in TCEP-free buffer, the dimeric form of the protein was purified from the mixture by SEC and analysed by DSF. The data for the monomeric form of cysteine mutants was obtained for the samples incubated in buffer containing TCEP and separated on SEC in the same way.

**Fig. 7:**
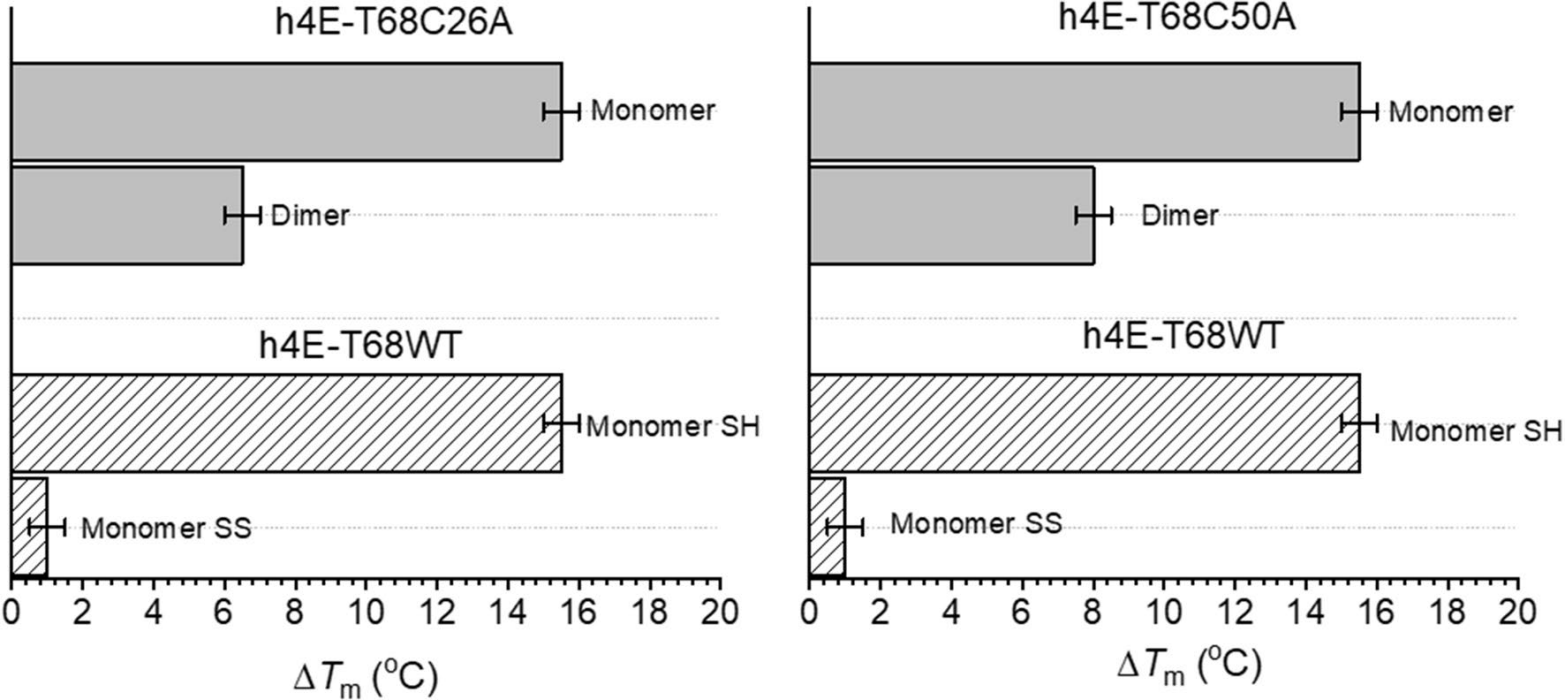
Influence of h4E-T(1–68) variants oligomerization on the thermal stability of eIF4E studied by DSF. The Δ*T*_m_ values were obtained as a difference in the *T*_m_ for 1:1 mixture of eIF4E with appropriate oligomeric form of h4E-T(1–68) variants and the for apo form of eIF4E. The concertation of eIF4E protein was 5 µM. The dimeric forms of C26A and C50A and monomeric SS form of WT h4E-T were investigated under non-reducing conditions, the monomeric forms of C26A and C50A and monomeric SH form of WT h4E-T under reducing conditions

Both dimers formed by the C26A and C50A mutants give a thermal shift of eIF4E (Δ*T*_m_) about two-fold lower than the corresponding monomer (Fig. 7), but about 2.5 °C lower than the previously investigated mixtures. However, the dimeric form stabilises eIF4E with the thermal shift ΔΔ*T*_m_ of 5.5 °C and 7 °C higher for C26A and C50A, respectively, as compared with the shift of the h4E-T(1–68) monomer with the intramolecular disulphide bond (monomer SS). These findings suggest that the intramolecular disulphide bond links together canonical and non-canonical 4E-binding motifs at the N-terminus of 4E-T and blocks the interaction between eIF4E and 4E-T proteins.

## Conclusions and implications

Cysteine is one of the least abundant amino acids in organisms and, together with tryptophan, one of the most highly conserved amino acids in proteins (Jordan et al. 2005; Passam and Chiu 2019). In addition, cysteine residue is frequently observed in the functional site of proteins, where it stabilises the structure by forming disulphide bonds, or performs catalytic and regulatory functions that are important for protein regulation during biological processes in cells (Marino and Gladyshev 2012). This versatility of the cysteine residue is largely due to high reactivity and chemical plasticity of its thiol group, whose chemical reactivity is strongly dependent on p*K*_a_ and can be influenced by the local environment (Marino and Gladyshev 2012; Passam and Chiu 2019).

Disulphide bonds have been observed in about one third of proteins present in the eukaryotic proteome (Hatahet and Ruddock 2009), and they can be classified into different groups based on their function. Structural disulphide bonds, which are important for stable protein conformation, are formed during protein folding in the endoplasmic reticulum (ER) and the mitochondrial intermembrane space (IMS) by specialised oxidoreductases (Kojer and Riemer 2014). Another type of disulphide bonds forms a catalytic motif in the active site of oxidoreductases, facilitating the formation and cleavage of disulphide bonds in substrate proteins during redox reactions. Recently, a new class of disulphide bonds has been defined, known as 'allosteric disulphide bonds', whose formation or cleavage at one site of a protein triggers a functional change at another site of the protein (Passam and Chiu 2019).

Formation of reversible disulphide bonds can be induced by reactive oxygen (ROS), nitrogen (RNS) or chloride (RCS) species. When the reactive cysteine thiols (R-SH/R-S^−^) encounter ROS, RNS or RCS, they can be oxidised to form highly reactive sulfenic acid (RSOH). Next, sulfenic acid can interact with nearby cysteine to form reversible intramolecular or intermolecular disulphide bonds, or form other irreversible oxidation products such as sulfinic or sulphonic acids (Cremers and Jakob 2013; Chio and Tuveson 2017). Importantly for this class of disulphide bonds, they are typically formed in the reducing environment of the cytosol (Cremers and Jakob 2013).

In order to adapt to oxidative stress and survive, cells use various translational control elements to reprogram gene expression (Hayes et al. 2020; Campos-Melo et al. 2021). Increased ROS levels trigger the assembly of stress granules (SGs), ribonucleoprotein complexes composed of stalled translation initiation complexes as well as host RNA binding proteins and other signalling proteins to promote cell survival (Panas et al. 2016; Ivanov et al. 2019; Riggs et al. 2020; Hofmann et al. 2021). Other cellular foci P-bodies (PBs), of which 4E-T is a major component and where 4E-T interacts with eIF4E, are present in every cell under physiological conditions. However, it has been shown that P-bodies increase in number and size under certain oxidative stresses (Andrei et al. 2005; Kamenska et al. 2014b; Riggs et al. 2020).

Human 4E-T protein, unlike its homologues from other eukaryotic organisms, has an additional cysteine within its N-terminal unstructured region responsible for eIF4E binding. The conserved cysteine residue (Cys50) in 4E-T is located close to the non-canonical eIF4E binding motif (NC motif), whereas the non-conserved cysteine (Cys26) is close to the canonical eIF4E binding motif (C motif). Our in vitro studies show that under non-reducing conditions, Cys26 forms an intramolecular disulfide bond with Cys50, linking the canonical and non-canonical eIF4E binding motifs at the N-terminus of 4E-T and blocking the interaction between the two proteins. These results suggest that intramolecular disulphide bond formation at 4E-T may act as a switch to regulate its interaction with eIF4E during oxidative stress. However, further in vivo studies are required to demonstrate that elevated levels of ROS can induce oxidant-mediated disulphide bond formation at 4E-T and regulate its interaction with eIF4E in the cell. Our findings undoubtedly highlight the need to control reducing conditions during in vitro studies of the human 4E-T protein.

### Supplementary Information

Below is the link to the electronic supplementary material.

Supplementary file1 (DOCX 124 KB)

## References

[CR1] Andrei MA, Ingelfinger D, Heintzmann R (2005). A role for eIF4E and eIF4E-transporter in targeting mRNPs to mammalian processing bodies. RNA.

[CR3] Bioradiations (2019) Protein thermal shift assays made easy with bio-rad’s family of CFX real-time PCR detection systems. https://www.bioradiations.com/protein-thermal-shift-assays-made-easy-with-bio-rads-family-of-cfx-real-time-pcr-detection-systems/. Accessed 13 Nov

[CR2] Borden KLB (2016). The eukaryotic translation initiation factor eIF4E wears a “ cap” for many occasions. Translation.

[CR4] Brandmann T, Fakim H, Padamsi Z (2018). Molecular architecture of LSM 14 interactions involved in the assembly of mRNA silencing complexes. EMBO J.

[CR5] Brown JI, Page BDG, Frankel A (2020). The application of differential scanning fluorimetry in exploring bisubstrate binding to protein arginine *N*-methyltransferase 1. Methods.

[CR6] Campos-Melo D, Hawley ZCE, Droppelmann CA, Strong MJ (2021). The integral role of RNA in stress granule formation and function. Front Cell Dev Biol.

[CR7] Chio IIC, Tuveson DA (2017). ROS in cancer: the burning question. Trends Mol Med.

[CR8] Cremers CM, Jakob U (2013). Oxidant sensing by reversible disulfide bond formation. J Biol Chem.

[CR9] Culjkovic B, Topisirovic I, Skrabanek L (2005). eIF4E promotes nuclear export of cyclin D1 mRNAs via an element in the 3 J UTR. J Cell Biol.

[CR10] Dostie Â, Ferraiuolo M, Pause A (2000). A novel shuttling protein, 4E-T, mediates the nuclear import of the mRNA 5’ cap-binding protein, eIF4E. EMBO J.

[CR11] Ferraiuolo MA, Basak S, Dostie J (2005). A role for the eIF4E-binding protein 4E-T in P-body formation and mRNA decay. J Cell Biol.

[CR12] Gao K, Oerlemans R, Groves MR (2020). Theory and applications of differential scanning fluorimetry in early-stage drug discovery. Biophys Rev.

[CR13] Gruner S, Peter D, Weber R (2016). The structures of eIF4E-eIF4G complexes reveal an extended interface to regulate translation initiation. Mol Cell.

[CR14] Hatahet F, Ruddock LW (2009). Protein disulfide isomerase: a critical evaluation of its function in disulfide bond formation. Antioxidants Redox Signal.

[CR15] Hayes JD, Dinkova-Kostova AT, Tew KD (2020). Oxidative stress in cancer. Cancer Cell.

[CR16] Hernández G (2022). The versatile relationships between eIF4E and eIF4E-interacting proteins. Trends Genet.

[CR17] Ho JJD, Lee S (2016). A cap for every occasion: alternative eIF4F complexes. Trends Biochem Sci.

[CR18] Hofmann S, Kedersha N, Anderson P, Ivanov P (2021). Molecular mechanisms of stress granule assembly and disassembly. Biochim Biophys Acta Mol Cell Res.

[CR19] Igreja C, Peter D, Weiler C, Izaurralde E (2014). 4E-BPs require non-canonical 4E-binding motifs and a lateral surface of eIF4E to repress translation. Nat Commun.

[CR20] Ivanov P, Kedersha N, Anderson P (2019). Stress granules and processing bodies in translational control. Cold Spring Harb Perspect Biol.

[CR21] Jackson RJ, Hellen CUT, Pestova TV (2010). The mechanism of eukaryotic translation initiation and principles of its regulation. Nat Rev Mol Cell Biol.

[CR22] Jordan IK, Kondrashov FA, Adzhubei IA (2005). A universal trend of amino acid gain and loss in protein evolution. Nature.

[CR23] Kamenska A, Lu WT, Kubacka D (2014). Human 4E-T represses translation of bound mRNAs and enhances microRNA-mediated silencing. Nucleic Acids Res.

[CR24] Kamenska A, Simpson C, Standart N (2014). eIF4E-binding proteins: new factors, new locations, new roles. Biochem Soc Trans.

[CR25] Kamenska A, Simpson C, Vindry C (2016). The DDX6–4E-T interaction mediates translational repression and P-body assembly. Nucleic Acids Res.

[CR26] Kinkelin K, Veith K, Grunwald M, Bono F (2012). Crystal structure of a minimal eIF4E-Cup complex reveals a general mechanism of eIF4E regulation in translational repression. RNA.

[CR56] Kojer K, Riemer J (2014). Balancing oxidative protein folding: the influences of reducing pathways on disulfide bond formation Biochimica et Biophysica Acta (BBA). Proteins Proteomics.

[CR27] Konarska MM, Padgett RASP (1984). Recognition of cap structure in splicing in vitro of mRNA. Cell.

[CR28] Krishna SN, Luan CH, Mishra RK (2013). A fluorescence-based thermal shift assay identifies inhibitors of mitogen activated protein kinase kinase 4. PLoS ONE.

[CR29] Kubacka D, Kamenska A, Broomhead H (2013). Investigating the consequences of eIF4E2 (4EHP) interaction with 4E-transporter on its cellular distribution in HeLa cells. PLoS ONE.

[CR30] Laue TM, Shah B, Ridgeway TM, Pelletier SL, Harding SE, Horton JC, Rowe AJ (1992). Computer-aided interpretation of sedimentation data for proteins. Analytical ultracentrifugation in biochemistry and polymer science.

[CR31] Li Y, Kiledjian M (2010). Regulation of mRNA decapping. Wiley Interdiscip Rev RNA.

[CR32] Ling SHM, Qamra R, Song H (2011). Structural and functional insights into eukaryotic mRNA decapping. Wiley Interdiscip Rev RNA.

[CR33] Luo Y, Na Z (2018). P-bodies: composition, properties, and functions. Biochemistry.

[CR34] Mader S, Lee H, Pause A, Sonenberg N (1995). The translation initiation factor eIF-4E binds to a common motif shared by the translation factor eIF-4 gamma and the translational repressors 4E-binding proteins. Mol Cell Biol.

[CR35] Marino SM, Gladyshev VN (2012). Analysis and functional prediction of reactive cysteine residues. J Biol Chem.

[CR36] Merrick WC (2015). eIF4F: a retrospective. J Biol Chem.

[CR37] Niesen FH, Berglund H, Vedadi M (2007). The use of differential scanning fluorimetry to detect ligand interactions that promote protein stability. Nat Protoc.

[CR38] Osborne MJ, Borden KLB (2015). The eukaryotic translation initiation factor eIF4E in the nucleus: taking the road less traveled. Immunol Rev.

[CR39] Ozgur S, Filipowicz W, Conti E (2015). Structure of a human 4E-T/DDX6/CNOT1 complex reveals the different interplay of DDX6-binding proteins with the CCR4-NOT complex. Cell Rep.

[CR40] Pace CN, Vajdos F, Fee L (1995). How to measure and predict the molar absorption coefficient of a protein. Protein Sci.

[CR41] Panas MD, Ivanov P, Anderson P (2016). Mechanistic insights into mammalian stress granule dynamics. J Cell Biol.

[CR42] Passam FJ, Chiu J (2019). Allosteric disulphide bonds as reversible mechano-sensitive switches that control protein functions in the vasculature. Biophys Rev.

[CR43] Patzelt E, Hartmuth K, Blaas D, Kuechler E (1987). Assembly of pre-mRNA splicing complex is cap dependent nucleic. Nucleic Acids Res.

[CR44] Peter D, Igreja C, Weber R (2015). Molecular architecture of 4E-BP translational inhibitors bound to eIF4E. Mol Cell.

[CR45] Rajpal G, Arvan P (2013). Handbook of biologically active pepetides disulfide bond formation.

[CR46] Räsch F, Weber R, Izaurralde E, Igreja C (2020). 4E-T-bound mRNAs are stored in a silenced and deadenylated form. Genes Dev.

[CR47] Richter JD, Sonenberg N (2005). Regulation of cap-dependent translation by eIF4E inhibitory proteins. Nature.

[CR48] Riggs CL, Kedersha N, Ivanov P, Anderson P (2020). Mammalian stress granules and P bodies at a glance. J Cell Sci.

[CR49] Scheuermann TH, Brautigam CA (2015). High-precision, automated integration of multiple isothermal titration calorimetric thermograms: new features of NITPIC. Methods.

[CR50] Schuck P (2000). Size-distribution analysis of macromolecules by sedimentation velocity ultracentrifugation and lamm equation modeling. Biophys J.

[CR51] Schuck P (2003). On the analysis of protein self-association by sedimentation velocity analytical ultracentrifugation. Anal Biochem.

[CR52] Senisterra G, Chau I, Vedadi M (2012). Thermal denaturation assays in chemical biology. Assay Drug Dev Technol.

[CR53] Sonenberg N, Hinnebusch AG (2009). Regulation of translation initiation in eukaryotes: mechanisms and biological targets. Cell.

[CR54] Xue B, Dunbrack RL, Williams RW (2010). PONDR-FIT: a meta-predictor of intrinsically disordered amino acids. Biochim Biophys Acta Proteins Proteomics.

[CR55] Zhao H, Piszczek G, Schuck P (2015). SEDPHAT—a platform for global ITC analysis and global multi-method analysis of molecular interactions. Methods.

