# Supplementary material for: An intramolecular disulphide bond in human 4E-T affects its binding to eIF4E1a protein
Source: Eur Biophys J. 2023 Oct 5;52(6-7):497–510. doi: 10.1007/s00249-023-01684-7 (PMC10618305; doi:10.1007/s00249-023-01684-7)
Supplement: Supplementary file 1 — Supplementary file1 (DOCX 124 KB) [file 249_2023_1684_MOESM1_ESM.docx]

**Supplementary Information**

Intramolecular disulphide bond in human 4E-T affects its binding to eIF4E1a protein

Joanna Zuberek^1^*, Marek Warzecha^1^, Mateusz Dobrowolski^1^, Anna Modrak-Wojcik^1^

^1^Division of Biophysics, Institute of Experimental Physics, Faculty of Physics, University of Warsaw, Poland

* corresponding author: Joanna.Zuberek@fuw.edu.pl

Joanna Zuberek ORCID:0000-0003-4798-2666

Anna Modrak-Wojcik: ORCID: 0000-0003-2616-2879

**Table S1.** Oligonucleotides used in this study to obtain constructs and mutants of 4E-T(1-68) peptide.

| **Oligonucleotide name** | **Name of the construct** | **Oligonucleotides sequences** |
| --- | --- | --- |
| h4ET68NdeI_Fwd | pET28a_ h4ET68 | 5’ AGACGCATATGGATAGGAGAAGT 3’ |
| h4ET68NdeI_Rev |  | 5’ AGTCTCGAGTTAGGCATGCCACTTCTC 3’ |
| h4ET68C26A_Fwd | pET28a_ h4ET68C26A | 5’GCCTCCTGCCTCCAAAGCCCCCCATCGCTATAC 3’ |
| h4ET68C26A_Rev |  | 5’GTATAGCGATGGGGGGCTTTGGAGGCAGGAGGC 3’ |
| h4ET68C50A_Fwd | pET28a_ h4ET68C50A | 5’CAAACAGAGGCCTTCAGCCCTTTCTGAAAAATATGACAG 3’ |
| h4ET68C50A_Rev |  | 5’CTGTCATATTTTTCAGAAAGGGCTGAAGGCCTCTGTTTGG3’ |

**Table S2.** Thermodynamic parameters for interaction human eIF4E1a with 4E-T(1-68) variants in the absence or presence 1 mM TCEP.

| **Variant of h4ET protein** |  | **Interaction h4ET variant with heIF4E1a**  **ITC experiments** | | | |
| --- | --- | --- | --- | --- | --- |
|  | **TCEP** | ***K*_as_ (µM^-1^)** | **Δ*G*^o^ (kcal/mol)** | **Δ*H* (kcal/mol)** | ***T*Δ*S* (kcal/mol)** |
|  |  |  |  |  |  |
| WT | - | 1.25 ± 0.15 | -8.18 ± 0.07 | -14.94 ± 0.31 | -6.76 ± 0.32 |
|  | + | 373 ± 68 | -11.49 ± 0.11 | -38.7 ± 1.7 | -27.2 ± 1.2 |
| C26A | - | ~100 |  |  |  |
|  | + | 664 ± 84 | -11.82 ± 0.07 | -36.0 ± 0.7 | -24 2 ± 1.3 |
| C26AC50A | - | 327 ± 22 | -11.41 ± 0.04 | -37.7 ± 0.6 | -26.2 ± 0.6 |

**
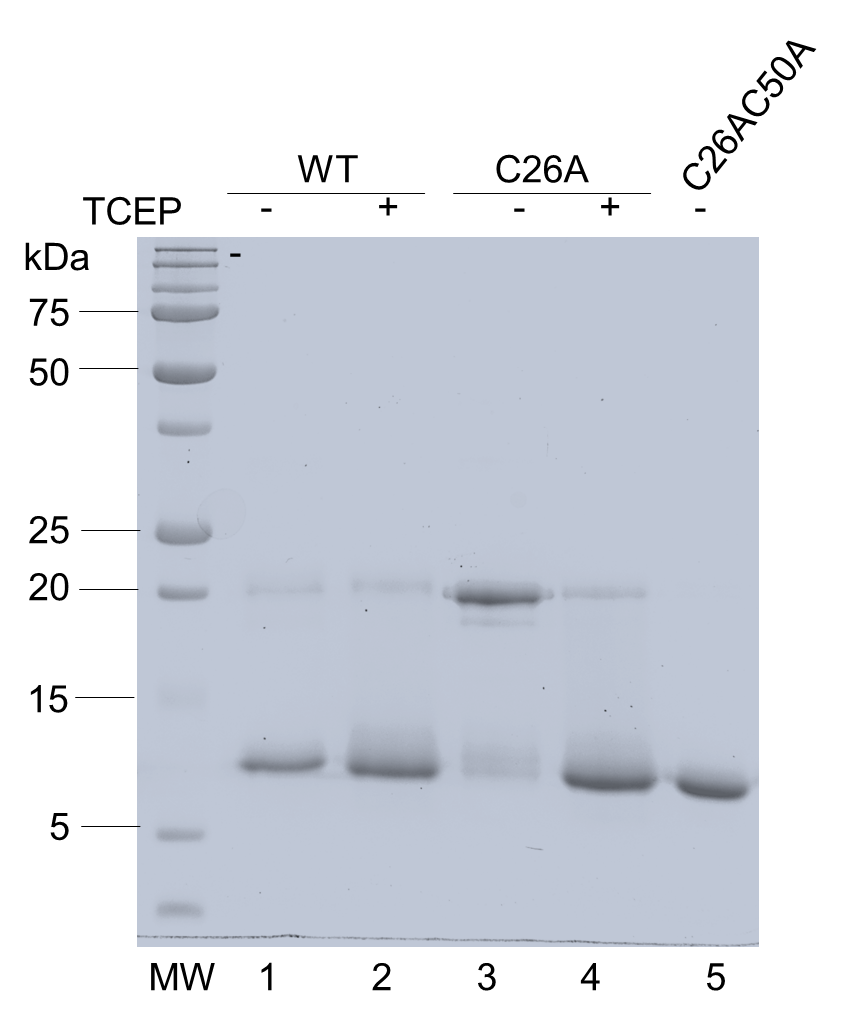
**

**Figure S1.** SDS-PAGE analysis of the fractions of h4E-T(1-68) variants purified on size exclusion chromatography and incubated by 24 hours with or without TCEP. MW, protein marker weight standards (Bio-Rad); lane 1, h4E-T(1-68)WT incubated without TCEP, lane 2, h4E-T(1-68)WT incubated with TCEP; lane 3, h4E-T(1-68)C26A incubated without TCEP; lane 4, h4E-T(1-68)C26A incubated with TCEP; lane 5, h4E-T(1-68)C26AC50A incubated without TCEP
